# Association of RNASEL and 8q24 variants with the presence and aggressiveness of hereditary and sporadic prostate cancer in a hispanic population

**DOI:** 10.1111/jcmm.12171

**Published:** 2013-11-14

**Authors:** Ignacio F San Francisco, Pablo A Rojas, Verónica Torres-Estay, Susan Smalley, Javier Cerda-Infante, Viviana P Montecinos, Claudia Hurtado, Alejandro S Godoy

**Affiliations:** a Departamento de Urología Facultad de Medicina, Pontificia Universidad Católica de ChileSantiago, Chile; b Departamento de Ciencias Fisiológicas Facultad de Ciencias Biológicas, Pontificia Universidad Católica de ChileSantiago, Chile; c Departamento de Nutrición, Metabolismo y Diabetes Facultad Medicina, Pontifica Universidad Católica de ChileSantiago, Chile; d Departamento de Hemato-Oncología Facultad de Medicina, Pontificia Universidad Católica de ChileSantiago, Chile; e Laboratorio de Oncología y Genética Molecular, Clínica Las CondesSantiago, Chile; f Department of Urology, Roswell Park Cancer InstituteBuffalo, NY, USA

**Keywords:** rs6983267, 8q24, RNASEL, prostate cancer, polymorphism

## Abstract

To study the association between the polymorphisms Arg462Gln and Asp541Glu from the RNASEL gene (1q25), and the polymorphisms rs620861, rs1447295, rs6983267, rs7837328 from the chromosome 8q24 with the risk of presenting prostate cancer (PCa) and its clinical characteristics in a Hispanic (Chilean) population. The study was performed on 21 control patients and 83 patients diagnosed with PCa. Polymorphisms were analysed from blood samples through real-time PCR by using TaqMan probes, and the genetic analysis was performed with the SNPStats program. Also, a comparison was performed between clinical characteristics of PCa and the presence of the different polymorphism genotypes by using the Minitab software. There was a significant association between the genotype G/G from the polymorphism rs6983267 with an overall increased risk of PCa, in patients both with or without family history of PCa (OR = 4.47, 95% CI = 1.05–18.94, *P* = 0.034 and OR = 3.57, 95% CI = 0.96–13.35, *P* = 0.037, respectively). Regarding clinical parameters, patients carrying the genotype C/C from the polymorphism Asp541Glu had significantly higher prostate-specific antigen (PSA) levels than patients carrying the other genotypes (*P* = 0.034). Moreover, patients with the genotype G/G of rs6983267 had higher PSA levels (*P* = 0.024). The polymorphism rs6983267 from region 3 of the chromosome 8q24 appears to be a prominent risk factor for PCa and a biomarker for cancer aggressiveness in the group of patients who presented higher levels of PSA at the time of diagnosis.

## Introduction

Prostate cancer (PCa) is the most common non-cutaneous cancer and the second leading cause of male deaths from cancer in the United States 1. In Chile, according to the latest Health Ministry statistics published in 2012, PCa became the second leading cause of cancer-related deaths in men in 2008, with a rate of 20.2 per 100,000 inhabitants, surpassing lung cancer, and has only been outrun by gastric cancer 2.

Some recurrence and survival predictors have been identified in patients treated surgically or with radiation therapy for PCa. These predictors include the degree of differentiation of cancer (Gleason score), pre-operative prostate-specific antigen (PSA) levels, clinical stage and tumour volume in the needle biopsy specimen of radical prostatectomy. These predictors are the results of the biological behaviour of PCa because of its molecular and genetic alterations 3–4; however, to our knowledge, no genetic predictors have been proved to be associated with aggressiveness in PCa, despite having genes associated with PCa.

The RNASEL gene (1q24-25) was first described in 1996 by Smith *et al*. 5 and has been proposed as a candidate gene for the HPCa1 (Hereditary Prostate Cancer 1) locus. RNASEL is a ribonuclease that degrades viral RNA and regulates cellular apoptosis 6. RNASEL gene mutations have been identified in SPCa (Sporadic Prostate Cancer) and HPCa 5–6; nonetheless, there is strong evidence supporting the importance of RNASEL to HPCa 7–8. At least 11 single-nucleotide polymorphisms (SNPs) and mutations have been associated with both SPCa and HPCa. However, the majority of these studies have been performed in Nordic, white Anglo-Saxon, African-American and Asian populations, and only a few in Hispanic population. Rokman *et al*. 8 demonstrated an association between the SNP Arg462Gln and HPCa in Finnish patients and found no association in patients with no family history of PCa. Additionally, the authors showed no difference between controls and patients when the presence of Ile97Leu and Glu541Asp variants was assessed. In the case of the SPCa group, the authors found no differences in the presence of the three polymorphisms (Ile97Leu, Arg462Gln, and Asp541Glu) when compared with the control group.

To our knowledge, few studies have been published regarding RNASEL in Hispanic population. Shook *et al*. 6 found that the presence of the variant Arg462Gln increased four times (11% *versus* 3%) the risk of PCa in a Hispanic population. The same authors demonstrated an association of the polymorphisms Arg462Gln and Asp541Glu with the risk of PCa (OR: 3.92, *P* = 0.004, and OR: 1.72, *P* = 0.030, respectively).

Chromosome 8 has been implicated in colon and breast cancer and, in the last few years, it has been associated also with the risk of PCa. Zheng *et al*. 9 studied men with PCa in the Swedish population and showed that the SNP rs1447295 from region 1 of chromosome 8q24 was one of the most strongly associated with the PCa risk, but not with the aggressiveness of PCa. In 2009, Al Olama *et al*. 10 identified eight new SNPs in 8q24, in a Caucasian population. Each SNP was independently associated with PCa, highlighting a new SNP (rs620861). However, these SNPs were not associated with cancer aggressiveness. Yeager *et al*. 11, in the same issue of this journal, indicated that rs620861 was strongly associated with PCa risk in a Caucasian specimen as well. In 2009, Penney *et al*. 12 studied over a thousand cases and controls by using eight known SNPs, focusing their association analysis in cancer aggressiveness and mortality. They found that the presence of SNPs, especially rs1447295, was associated with diagnosis at an earlier age, but not with cancer aggressiveness. In this study, only one of the SNPs (rs6983267) associated with elevated levels of PSA. In the only study performed in Hispanic population, Beuten *et al*. 13 analysed 49 SNPs in the 8q24 region. Of the SNPs analysed, 12 were associated with the risk of PCa, with rs7837328 and rs921146 being the most important and showing independent effects (OR: 2.55, 95% CI: 1.51–4.31, *P* = 4.33 × 10^−4^, and OR: 2.09, 95% CI: 1.40–3.12, *P* = 3.13 × 10^−4^, respectively). However, in this study, the authors did not investigate the association of SNPs with the degree of cancer aggressiveness or PSA levels.

In summary, there is ample evidence to suggest an association between SNPs from both the RNASEL gene and the chromosome 8q24 with the risk of PCa as in its clinical characteristics. To test this hypothesis, we selected SNPs Arg462Gln and Asp541Glu from the RNASEL gene; and SNPs rs7837328, rs1447295, rs620861, and rs6983267 from chromosome 8q24 to be studied in a Hispanic population of patients with sporadic or hereditary PCa. The objective of this study was to determine the association between SNPs from RNASEL and chromosome 8q24 with the risk of PCa, and its aggressiveness, in a Hispanic (Chilean) population.

## Materials and methods

### Study population

The population of the study included 83 patients diagnosed with PCa from two clinical centres: the Clinical Hospital of Pontificia Universidad Católica de Chile (PUC) and the Sótero del Río Hospital, from Santiago, Chile. Twenty-one men over 40 years old without a PCa diagnosis (PSA <4 ng/ml and normal Digital Rectal Exam- DRE) were used as a control group according to the literature that has shown that patients with PSA levels less than 4 ng/ml and normal DRE have a lower incidence of PCa 14 than patients in whom a prostate needle biopsy is performed 15–18. Patients with PCa were diagnosed by using the needle biopsy procedure. Of the 83 patients, 15 were classified as HPCa according to Carter *et al*. 19, like for example, when three or more family members have had PCa or when two family members were younger than 55 at the time of diagnosis. Clinical and pathological variables such as age at diagnosis, PSA at diagnosis, tumour volume, Gleason Score of the surgical specimen and histopathological stage were assessed. The study was approved by the ethics committee of the Clinical Hospital of the Pontificia Universidad Católica de Chile and the ethics committee of the Sótero del Río Hospital. All patients signed an informed consent.

### Extraction and quantification of DNA

The DNA was extracted from the ‘buffycoat’, with the Qiagen extraction kit (QLAamp DNA Mini Blood Kit, Valencia, CA, USA) according to the manufacturer’s instructions. Quantification of the extracted DNA was performed with the Epoch micro-volume spectrophotometric system from BioTek (Winooski, VT, USA).

### Genetic analysis

The polymorphisms were analysed through Genotyping testing with Taqman probes. The following compositions were used for each test: 20 ng of genomic DNA, 1× of TaqManGenotyping Master Mix and 1× TaqMan® Pre-Designed SNP GenotypingAssays, conducting the tests in the StepOne equipment of Applied Biosystem (Foster City, CA, USA).

### Statistical analysis

Hardy–Weinberg equilibrium was verified in the controls. Allele frequencies were estimated in cancer specimens and controls through gene counting by using the online SNPstats program 20. Dominant, recessive and overdominant models were used to establish the association of the SNPs with PCa, by using the model with the lowest value of the Akaike information criterion (AIC) 21. Fisher’s Exact Test was used to detect statistical differences between cancer specimens and controls in the genotype frequency of polymorphisms. Odd ratios (ORs) and 95% confidence intervals were computed by using Binary Logistic Regression. The association analysis between the presence of SNPs and clinical and pathological PCa patient characteristics was performed as follows: one-way anova was used to analyse the age at diagnosis. Prostate-specific antigen and tumour volume data were analysed by using the Kruskal–Wallis nonparametric test for medians. The frequency of HPCa and tumour stage was analysed by using the Fisher’s Exact Test for 2 × 3 contingency tables. Statistical analysis was performed with the statistical software Minitab®.

## Results

### Distribution of alleles and genotypes of the polymorphisms

Genotyping was performed on a total of 104 patients: 68 with SPCa, 15 with HPCa and 21 controls. The SNPs Arg462Gln and Asp541Glu from the RNASEL gene and the SNPs rs7837328, rs1447295, rs620861 and rs6983267 from chromosome 8q24 were analysed in all of the patients. The distribution of genotypes and alleles of RNASEL polymorphisms and on chromosome 8q24 is shown in Table 1. It was noted that the most frequent allele for Arg462Gln was C, in both the cancer group (70%) and the control group (67%), with C/C being the most frequent genotype for the same SNP. The most frequent allele for Asp541Glu was A in both groups (cancer 60%, control 55%), while A/A was the most frequent genotype. The most frequent allele for rs620861 was G with a frequency of 69% in both the cancer group and the control group. The most frequent genotype in both groups was G/G. For the SNP rs1447295, the most frequent allele was C with frequency of 81% in the cancer group and 86% in the control group, with the genotype C/C being the most common. The most frequent allele for SNP rs7837328 in both groups was G (cancer 63%, control 67%) with genotype G/A being the most frequent. SNP rs6983267 represents a special situation; while allele G was the most common in the cancer group (57%), allele T was the most common in the control group (55%). However, there was no statistical association between these alleles and cancer (*P* = 0.22, data not shown in table). Analysis of the genotypes showed that G/T was the most common genotype in both groups.

**Table 1:** Allele and genotype frequencies in cancer and control groups

SNP	Allele/Genotype	Total (proportion)	Cancer (proportion)	Control (proportion)
Arg462Gln	C	145 (0.7)	117 (0.7)	28 (0.67)
	T	63 (0.3)	49 (0.3)	14 (0.33)
	C/C	54 (0.52)	43 (0.52)	11 (0.52)
	C/T	37 (0.36)	31 (0.37)	6 (0.29)
	T/T	13 (0.12)	9 (0.11)	4 (0.19)
Asp541Glu	A	122 (0.59)	99 (0.6)	23 (0.55)
	C	86 (0.41)	67 (0.4)	19 (0.45)
	A/A	41 (0.39)	34 (0.41)	7 (0.33)
	A/C	40 (0.38)	31 (0.37)	9 (0.43)
	C/C	23 (0.22)	18 (0.22)	5 (0.24)
rs620861	G	143 (0.69)	114 (0.69)	29 (0.69)
	A	65 (0.31)	52 (0.31)	13 (0.31)
	A/A	7 (0.07)	6 (0.07)	1 (0.05)
	G/A	51 (0.49)	40 (0.48)	11 (0.52)
	G/G	46 (0.44)	37 (0.45)	9 (0.43)
rs1447295	C	171 (0.82)	135 (0.81)	36 (0.86)
	A	37 (0.18)	31 (0.19)	6 (0.14)
	A/A	5 (0.05)	4 (0.05)	1 (0.05)
	C/A	27 (0.26)	23 (0.28)	4 (0.19)
	C/C	72 (0.69)	56 (0.67)	16 (0.76)
rs6983267	G	112 (0.54)	93 (0.57)	19 (0.45)
	T	94 (0.46)	71 (0.43)	23 (0.55)
	G/G	33 (0.32)	30 (0.37)	3 (0.14)
	G/T	46 (0.45)	33 (0.4)	13 (0.62)
	T/T	24 (0.23)	19 (0.23)	5 (0.24)
rs7837328	G	131 (0.63)	103 (0.62)	28 (0.67)
	A	77 (0.37)	63 (0.38)	14 (0.33)
	A/A	11 (0.11)	9 (0.11)	2 (0.1)
	G/A	55 (0.53)	45 (0.54)	10 (0.48)
	G/G	38 (0.37)	29 (0.35)	9 (0.43)

### Association between SNPs and PCa

A comparison (represented in Table 2) was made between the cancer group and the control group to determine whether the genotype of each SNP was related to PCa. The study was based on the most suitable inheritance models for each SNP. When comparing the genotype G/G with the sum of the genotypes G/T + T/T from the SNP rs6983267, using the dominant model, an increased risk of presenting PCa was observed in the presence of the genotype G/G (OR = 3.46, 95% IC = 0.94–12.73, *P* = 0.039). The cancer group was divided into patients with HPCa or SPCa (according to the patient’s history) and then the two groups were compared with the control group to determine whether there was any association between the SNPs with the risk of PCa. The comparison between hereditary cancer group and the controls is shown in Table 3. Our results indicated that, when comparing the genotypes G/T with G/T + T/T from the SNP rs6983267, in an overdominant model, the genotypes T/T and G/G increased the risk of HPCa (OR = 4.47, 95% IC = 1.05–18.94, *P* = 0.034). Moreover, when the sporadic cancer group was compared with the control group (Table 4), SNP rs6983267 was highlighted again. In this case, comparison of the genotype G/G with the sum of genotypes G/G + T/T indicated that G/G increased the risk of SPCa (OR = 3.57, 95% IC = 0.96–13.35, *P* = 0.037).

**Table 2:** Association of SNP with CaP as inheritance model

SNP	Model	Genotype	Cancer	Control	OR (CI 95%)	*P*-value
Arg462Gln	Recessive	C/C-C/T	74 (89.2%)	17 (81%)	1.93 (0.53–7.03)	0.33
		T/T	9 (10.8%)	4 (19.1%)		
Asp541Glu	Dominant	A/A	34 (41%)	7 (33.3%)	1.39 (0.51–3.80)	0.52
		A/C-C/C	49 (59%)	14 (66.7%)		
rs620861	Recessive	G/G-A/G	77 (92.8%)	20 (95.2%)	0.64 (0.07–5.64)	0.68
		A/A	6 (7.2%)	1 (4.8%)		
rs1447295	Dominant	C/C	56 (67.5%)	16 (76.2%)	0.65 (0.21–1.96)	0.43
		A/C-A/A	27 (32.5%)	5 (23.8%)		
rs6983267	Dominant	G/G	30 (36.6%)	3 (14.3%)	3.46 (0.94–12.73)	0.039
		G/T-T/T	52 (63.4%)	18 (85.7%)		
rs7837328	Dominant	G/G	29 (34.9%)	9 (42.9%)	0.72 (0.27–1.90)	0.5
		A/G-A/A	54 (65.1%)	12 (57.1%)		

**Table 3:** Association of SNPs with HPCa as inheritance model

SNP	Model	Genotype	Cancer	Control	OR (CI 95%)	*P*-value
Arg642Gln	Dominant	C/C	6 (40%)	11 (52.4%)	0.61 (0.16–2.32)	0.46
		C/T-T/T	9 (60%)	10 (47.6%)		
Asp541Glu	Recessive	A/A-A/C	10 (66.7%)	16 (76.2%)	0.63 (0.14–2.72)	0.53
		C/C	5 (33.3%)	5 (23.8%)		
rs620861	Recessive	G/G-A/G	15 (100%)	20 (95.2%)	NA (0.00–NA)	0.29
		A/A	0 (0%)	1 (4.8%)		
rs1447295	Dominant	C/C	10 (66.7%)	16 (76.2%)	0.63 (0.14–2.72)	0.53
		A/C-A/A	5 (33.3%)	5 (23.8%)		
rs6983267	Overdom	T/T-G/G	11 (73.3%)	8 (38.1%)	4.47 (1.05–18.94)	0.034
		G/T	4 (26.7%)	13 (61.9%)		
rs7837328	Recessive	G/G-A/G	11 (73.3%)	19 (90.5%)	3.45 (0.54–22.02)	0.21
		A/A	4 (26.7%)	2 (9.5%)		

**Table 4:** Association of SNPs with SPCa as inheritance model

SNP	Model	Genotype	Cancer	Control	OR (CI 95%)	*P*-value
Arg462Gln	Recessive	C/C-C/T	62 (91.2%)	17 (81%)	2.43 (0.62–9.61)	0.22
		T/T	6 (8.8%)	4 (19.1%)		
Asp541Glu	Dominant	A/A	29 (42.6%)	7 (33.3%)	1.49 (0.53–4.15)	0.44
		A/C-C/C	39 (57.4%)	14 (66.7%)		
rs620861	Recessive	G/G-A/G	62 (91.2%)	20 (95.2%)	0.52 (0.06–4.55)	0.52
		A/A	6 (8.8%)	1 (4.8%)		
rs1447295	Overdomi	C/C-A/A	49 (72.1%)	17 (81%)	0.61 (0.18–2.04)	0.4
		A/C	19 (27.9%)	4 (19.1%)		
rs6983267	Dominan	G/G	25 (37.3%)	3 (14.3%)	3.57 (0.96–13.35)	0.037
		G/T-T/T	42 (62.7%)	18 (85.7%)		
rs7837328	Overdomi	G/G-A/A	28 (41.2%)	11 (52.4%)	0.64 (0.24–1.70)	0.37
		A/G	40 (58.8%)	10 (47.6%)		

### Association between SNPs and clinical prostate cancer

A comparison was made between the variables: age at diagnosis and PSA levels at diagnosis, post-operative Gleason score, tumour volume and pathological stage (organ confined or not, according to TNM) between the different genotypes of the SNP studied. First, a comparison was made among the three genotypes of each SNP, as shown in Table 5. We found no association between the presence of SNP Arg462Gln and the variables analysed. However, our results indicated that, for the SNP Asp541Glu, genotype C/C presented significantly higher PSA level than genotypes A/C and A/A at the time of diagnosis (*P* = 0.034). Asp541Glu did not present any association with the other clinical parameters previously mentioned. Similar results were observed with the four SNPs on chromosome 8q24 (rs7837328, rs1447295, rs620861 and rs6983267). Neither of them was related to the variables described above. The same analysis was conducted to compare the genotypes according to the most suitable inheritance model (Table 6). This analysis indicated that genotype G/G from SNP rs6983267 was associated with a significantly higher level of PSA when compared with genotypes G/T and T/T (*P* = 0.024) at the time of diagnosis. Similarly, it was observed that for SNP Asp541Glu, genotypes A/C or C/C were significantly associated with organ-confined cancer when compared with genotype A/A (*P* = 0.041). A similar result was observed for rs620861, in which the genotypes G/G and A/G were significantly associated with organ-confined cancer when compared with genotype A/A (*P* = 0.027). The rest of the SNPs showed no significant differences when compared with the variables studied.

**Table 5:** Association of SNPs with clinical and pathological variables of CaP patients

	Asp541Glu				Arg462Gln			
	A/A	A/C	C/C	*P*	C/C	C/T	T/T	*P*
Age (years)	64	63	66	0.77	64	63	69	0.16
PSA (ng/ml)	8.32	6.64	9.1	0.03	8	7.5	10.91	0.49
Organ confined(n)	14	20	8	0.36	21	17	4	0.94
Gleason score	7	7	7	–	7	7	7	–
Tumour vol (cc)	6	3.1	4	0.45	3.82	3.4	4.77	0.48

	rs6983267				rs7837328			
	G/G	G/T	T/T	*P*	G/G	G/A	A/A	*P*
Age (years)	64	64	61	0.95	65	64	63	0.24
PSA (ng/ml)	8.6	6.78	7.42	0.1	8.1	7.84	8.2	0.88
Organ confined (n)	19	14	9	0.46	11	25	6	0.7
Gleason score	7	7	7	–	7	7	7	–
Tumour vol (cc)	2.59	6	2.1	0.33	3.1	4.1	2.78	0.94

	rs620861				rs1447295			
	G/G	A/G	A/A	*P*	C/C	C/A	A/A	*P*
Age (years)	65	64	67	0.16	64	65	61	0.6
PSA (ng/ml)	8.5	7.54	7.9	0.49	8.56	6.78	6.14	0.26
Organ confined (n)	16	20	6	0.94	27	13	2	0.38
Gleason score	7	7	7	–	7	7	7	–
Tumour vol. (cc)	7.1	2.9	2	0.48	3.2	4.33	6.7	0.97

PSA: prostate-specific antigen; tumour vol: tumour volume.

**Table 6:** Association of SNPs as inheritance model with clinical and pathological variables of CaP patients

	Arg462Gln (Recessive model)			Asp541Glu (Dominant model)		
	C/C-C/T	T/T	*P*	A/C-C/C	A/A	*P*
Age (years)	62.14	68	0.067	62.47	63.34	0.65
PSA (ng/ml)	11.01	11.62	0.88	11.88	12.15	0.93
Organ confined (n)	38	4	1.00	28	14	0.041
Gleason score	7	7	–	7	7	–
Tumour vol. (cc)	5.67	6.24	0.78	5.13	6.84	0.24

	rs620861 (Recessive model)			rs1447295 (Dominant model)		
	G/G-A/G	A/A	*P*	A/C-A/A	C/C	*P*
Age (years)	65.52	66.5	0.27	63.5	62.5	0.64
PSA (ng/ml)	12.29	8.6	0.52	8.68	13.37	0.17
Organ confined (n)	36	6	0.027	15	27	0.41
Gleason Score	7	7	–	7	7	–
Tumour vol.(cc)	5.89	4.24	0.51	5.79	5.72	0.96

	rs6983267 (Dominant model)			rs7837328 (Dominant model)		
	G/T-T/T	G/G	*P*	A/A-A/G	G/G	*P*
Age (years)	62.76	62.72	0.98	62.81	62.84	0.99
PSA (ng/ml)	8.76	14.66	0.024	12.72	10.44	0.5
Organ confined (n)	23	19	0.31	31	11	0.42
Gleason Score	7	7	–	7	7	–
Tumour vol. (cc)	6.04	5.32	0.61	5.78	5.66	0.93

PSA: prostate-specific antigen; tumour vol: tumour volume.

## Discussion

Up until now, little is known about the genetic mechanisms involved in the development, progression and aggressiveness of PCa. In this study, we provided insights into the association of SNPs from RNASEL gene and chromosome 8q24 with the risk of PCa and in a hispanic (Chilean) population. In this sense, it is difficult to compare our results with existing literature, as most previous studies refer to Caucasian, African or Asian population, while data regarding Hispanic population are insufficient.

One of the first findings that stands out from our results is that all studied SNPs were present in patients with cancer, regardless of whether it was a hereditary or sporadic cancer, as well as in control patients (without cancer). However, only some of the SNPs genotypes were statistically associated with the presence of cancer, and therefore, these SNPs could be considered as risk factors for PCa.

Regarding the SNPs from the RNASEL gene Arg462Gln and Asp541Glu, our study demonstrated no association with cancer, which differs from the study reported by Shook *et al*. 6. In this study, Hispanic patients with PCa demonstrated association of the polymorphisms Arg462Gln and Asp541Glu with the risk of PCa (OR: 3.92, *P* = 0.004, and OR: 1.72, *P* = 0.030, respectively). In agreement with our results, there are several reports in which the authors demonstrated no association between the SNPs from RNASEL and cancer. In fact, Wei *et al*. 22 published in 2011 a meta-analysis of 11 case–control studies with 4730 cancer patients and 4218 controls in total for Asp541Glu, and 17 case–control studies with 9337 cancer patients and 8921 controls for Arg462. In this meta-analysis, the authors found no association between Arg462Gln and Asp541Glu with the risk of presenting PCa. Thus, there is a need for developing more standardized studies to understand the association between RNASEL and PCa. Our results indicated that, in the case of Asp541Glu, genotype C/C showed a significantly higher level of PSA than the other genotypes. However, when comparing the aggressiveness of PCa measured by histopathological stage, we observed that genotype C/C and A/C showed a higher number of organ-confined cancer (less than pT3), which would reflects less aggressiveness for both genotypes, a finding that contrasts with the higher PSA level observed in genotype C/C. Nakazato *et al*. 23 reported that the wild-type homozygous genotype (Asp/Asp) from Asp541Glu was associated with an increased familial prostate cancer risk (OR = 7.37) in Japanese men; however, they did not find any association between Arg462Gln and Asp541Glu and pathological stage and grade.

Regarding the SNPs on chromosome 8q24, our results indicated that for rs6983267, the genotype G/G presented more risk of PCa (both hereditary and sporadic), a result that resembles the findings reported by Cornu *et al*. 24 in 2011. In this article, the authors showed that genotype G/G of rs6983267 was related to an increased incidence of PCa in younger Caucasian men, as was also reported by Lange *et al*. 25. Lange *et al*., found a significant association between rs6983267 and PCa in patients younger than 55. Additionally, in 2011, Papanikolopoulou *et al*. 26 reported a significant association between rs6983267 and PCa in a Greek population (OR = 2.84, *P* = 0.002). Similarly, Cheng *et al*. 27 found a significant association between rs6983267 and PCa. Moreover, these authors showed that the G allele was related to advanced PCa (TNM >T2c) (OR = 1.35, 95% CI: 1.12–1.63, *P* = 0.002). A recent meta-analysis performed by Troutman *et al*. 28 confirmed the association between rs6983267 and PCa. However, up until our work, and besides Beuten’s study 13, which reported outstanding results for rs6983267, there was no study in Hispanic population corroborating these findings. The mechanism by which rs6983267 of region 3 on chromosome 8q24 affects the risk of PCa is currently unknown, but the hypothesis is that SNP would enhance Wnt signalling, a key pathway for the induction of bone metastases in PCa 29.

In our study, the results also indicated that the G/G genotype from the SNP rs6983267 had significantly higher PSA than the other genotypes. This finding was also described by Penney *et al*. 12, who reported that, from the SNPs studied, only rs6983267 showed significantly higher levels of PSA. In the previously mentioned Papanikolopoulou’s study 26, there was no association between SNPs and clinical features, such as the PSA level. Meanwhile, in the Troutman *et al*. meta-analysis, the association between rs6983267 and clinical parameters of aggressiveness was inconsistent 28. Thus, genotype G/G of the SNP rs6983267 is related to cancer and is also associated with higher PSA, and has been indicated repeatedly in the latest publications as a risk factor for PCa. Validation of this SNP as a biomarker for PCa, however, needs further research. We observed that the genotypes G/G and A/G from the SNP rs620861 on chromosome 8q24 were associated with a higher number of organ-confined cancers when compared with the genotype A/A. Genotype A/A from the SNP rs620861 was related to an increased number of patients with extra-prostatic spread, which may be considered as a factor for aggressiveness in PCa. Other than being related to PCa 10,11, there is no further description in the literature about the relationship between the SNP rs620861 with PCa aggressiveness and therefore, our findings require further exploration.

In conclusion, our study indicates that the SNP rs6983267 from region 3 of chromosome 8q24, appear to be a prominent risk factor for PCa, and a marker for cancer aggressiveness when presenting PSA levels that are significantly higher at diagnosis. Therefore, more emphasis should be placed in the study and characterization of this SNP, primarily, in the Hispanic population.
